# Structure and Activity of *Streptococcus pyogenes* SipA: A Signal Peptidase-Like Protein Essential for Pilus Polymerisation

**DOI:** 10.1371/journal.pone.0099135

**Published:** 2014-06-09

**Authors:** Paul G. Young, Thomas Proft, Paul W. R. Harris, Margaret A. Brimble, Edward N. Baker

**Affiliations:** 1 School of Biological Sciences, University of Auckland, Auckland, New Zealand; 2 Department of Molecular Medicine, Faculty of Medical and Health Sciences, University of Auckland, Auckland, New Zealand; 3 School of Chemical Sciences, University of Auckland, Auckland, New Zealand; 4 Maurice Wilkins Centre for Molecular Biodiscovery, University of Auckland, Auckland, New Zealand; University of Texas-Houston Medical School, United States of America

## Abstract

The pili expressed on the surface of the human pathogen *Streptococcus pyogenes* play an important role in host cell attachment, colonisation and pathogenesis. These pili are built from two or three components, an adhesin subunit at the tip, a major pilin that forms a polymeric shaft, and a basal pilin that is attached to the cell wall. Assembly is carried out by specific sortase (cysteine transpeptidase) enzyme. These components are encoded in a small gene cluster within the *S. pyogenes* genome, often together with another protein, SipA, whose function is unknown. We show through functional assays, carried out by expressing the *S. pyogenes* pilus components in *Lactococcus lactis*, SipA from the clinically important *M1T1* strain is essential for pilus assembly, and that SipA function is likely to be conserved in all *S. pyogenes*. From the crystal structure of SipA we confirm that SipA belongs to the family of bacterial signal peptidases (SPases), which process the signal-peptides of secreted proteins. In contrast to a previous arm-swapped SipA dimer, this present structure shows that its principal domain closely resembles the catalytic domain of SPases and has a very similar peptide-binding cleft, but it lacks the catalytic Ser and Lys residues characteristic of SPases. In SipA these are replaced by Asp and Gly residues, which play no part in activity. We propose that SipA functions by binding a key component at the bacterial cell surface, in a conformation that facilitates pilus assembly.

## Introduction

*Streptococcus pyogenes* (Group A Streptococcus [GAS]) is a highly adapted human pathogen that readily infects and colonizes the pharynx or skin, giving rise to inflammatory conditions such as pharyngitis and erysipelas. Although mostly mild and readily treatable with antibiotics, some infections can lead to very severe invasive diseases such as necrotising fasciitis or streptococcal toxic shock syndrome [1]. Moreover, chronic infections can result in acute rheumatic fever and rheumatic heart disease, which is a major problem in developing countries and in select populations of developed countries such as the indigenous and Pacific Island communities in New Zealand and Australia [2], [3], [4].

Host-pathogen interactions require the adhesion of *S. pyogenes* to dermal and epithelial cells. Recently it has been discovered that GAS produces pili on its surface [5], and that these pili are instrumental in mediating attachment of GAS to host cells and disease development [6], [7]. Pili have also been implicated in biofilm formation, which is believed to help bacteria to survive and proliferate during the infection process [7]. GAS pili are encoded within the highly variable Fibronectin-binding, Collagen-binding, T antigen (FCT) gene cluster, which can be classified into 9 sub-types [8]. The most prevalent FCT types are FCT3 and FCT4, found in approximately 60% of isolates [9]. FCT3 and FCT4 cluster together with FCT2, which includes the highly pathogenic and clinically relevant M1/T1 serotype. [8], [9]. The core elements of FCT types 2, 3 and 4 encode genes for the structural proteins that make up the pili.

GAS pili usually consist of three components, a major pilin or backbone protein (BP, also known as FctA), which forms the polymeric shaft, and two minor pilin proteins or accessory pilins (AP1 and AP2, also known as Cpa and FctB, respectively, in FCT 3 and 4) [5]. The shaft is assembled by covalent polymerisation of successive BP molecules [5], [10], [11], while the minor pilin AP1 (Cpa) is the adhesin at the tip of the pilus [11], [12] and the basal pilin AP2 covalently links the pilus to the cell wall peptidoglycan [12], [13], [14]. The polymerisation of the pilin subunits is mediated by a specific sortase (SrtC), which catalyses the formation of isopeptide bonds between subunits [5], [10], [14]. In FCT types 2, 3 and 4, another gene, *sipA*, is clustered with the pilin and sortase genes, in a strictly conserved order (*cpa*, *sipA*, *fctA*, *srtC*, *fctB*) and its gene product SipA has been shown for several FCT3 strains to be essential for pilus polymerisation [15], [16].

In contrast to the well-established roles played by the other GAS pilus proteins, that of SipA is little understood. Its amino acid sequence shows it to be homologous with the bacterial Type-I signal peptidases and the crystal structure of a truncated form of SipA demonstrated that it shares the same basic fold [17]. Type-I signal peptidases (SPase-I) are ubiquitous and essential membrane-bound proteases that cleave the signal-peptide sequence from pre-proteins translocated through both the Sec and TAT dependent secretion pathways [18], [19]. These enzymes have a characteristic Ser-Lys catalytic dyad, in which the serine acts as the nucleophile and the amino group of lysine provides the general base that deprotonates the serine hydroxyl group [19]. Most bacteria have only one active signal peptidase, which is essential for growth and survival [20], [21], but, some Gram-positive bacteria have several signal peptidases that appear to have overlapping sequence specificities [22].

In addition, it is increasingly apparent that Gram-positive bacterial genomes encode other proteins that are predicted to share the SPase-I architecture but lack an identifiable Ser-Lys catalytic dyad [17], [23]. SipA falls into this category of 'inactive' peptidases. It has atypical sequence motifs at the sites of the catalytic serine and lysine residues of true signal peptidases (conserved SPase-I boxes B and D) [15] and fails to show any detectable peptidase activity against pre-Cpa or synthetic peptides encompassing potential substrate cleavage sites [16]. SipA homologues are also present in the pilus gene clusters of *Streptococcus dysgalactiae*, *Streptococcus mitis*, *Streptococcus oralis*, *Streptococcus sanguinis* [24] and in some, but not all, *Streptococcus agalactiae* [25] and *Streptococcus pneumoniae* [26] pilus gene clusters. Interestingly, *Streptococcus suis* and *S. agalactiae* contain a SipA homologue, which in *S. suis* was found to be highly upregulated when bacteria interact with porcine brain microvascular endothelial cells [27], [28], and which appears to have an intact catalytic dyad.

Despite the essential role of SipA in FCT3 strain pilus polymerisation, little is known about how it participates in this process, whether it functions as pilus-specific peptidase but with alternative catalytic residues or has some non-enzymatic role as a chaperone or a component in some larger cell-surface assembly. We previously solved the crystal structure of a truncated form of SipA, which showed that it does indeed share the SPase-I fold [17]. This truncated form was found to form a domain-swapped dimer, however, in which the structure of the N-terminal region was disrupted; no peptide-binding groove was apparent and the strand that would normally carry the SPase-I catalytic serine was disordered.

Here we describe the crystal structure of a SipA molecule that comprises the complete extracellular portion of the protein. This shows that SipA does indeed have a peptide-binding groove very like that of *E. coli* SPase-I, but that it lacks the catalytic apparatus typical of signal peptidases. We show that it has no peptidase activity, but that it is essential for polymerisation of pilin subunits in FCT2 pili, and that its function is likely to be conserved in all strains of *S. pyogenes* that carry a SipA homologue.

## Results

### Structure determination

Initial attempts to determine the structure of Spy0127, the SipA protein from the serotype M1/T1 strain SF370, were unsuccessful as all constructs produced insoluble protein in *E. coli*. However, soluble SipA was purified from a local GAS strain 90/360S, typed as serotype T9 with a gene organization similar to that of the M9 strain 2720 [8], [13]. The T9 SipA structure (SipA_36-173_) solved in this study comprises the entire extracellular region of the protein, residues 36–173. The first 35 residues, encompassing the transmembrane anchor, were deleted to permit soluble expression.

SipA was initially purified from *E. coli* as a large soluble aggregate (>690 kDa) that eluted in a broad peak at the void volume during Size Exclusion Chromatography. This could be converted by the addition of glycine to a smaller, uniform species estimated to be 150 kDa; we speculate that the addition of glycine triggered some refolding or reorganisation. This purified 'reorganised' SipA is stable without glycine and is an octamer, as determined by size exclusion chromatography and dynamic light scattering (data not shown), and confirmed by the small angle X-ray scattering (SAXS) analysis.

**Figure 1:**
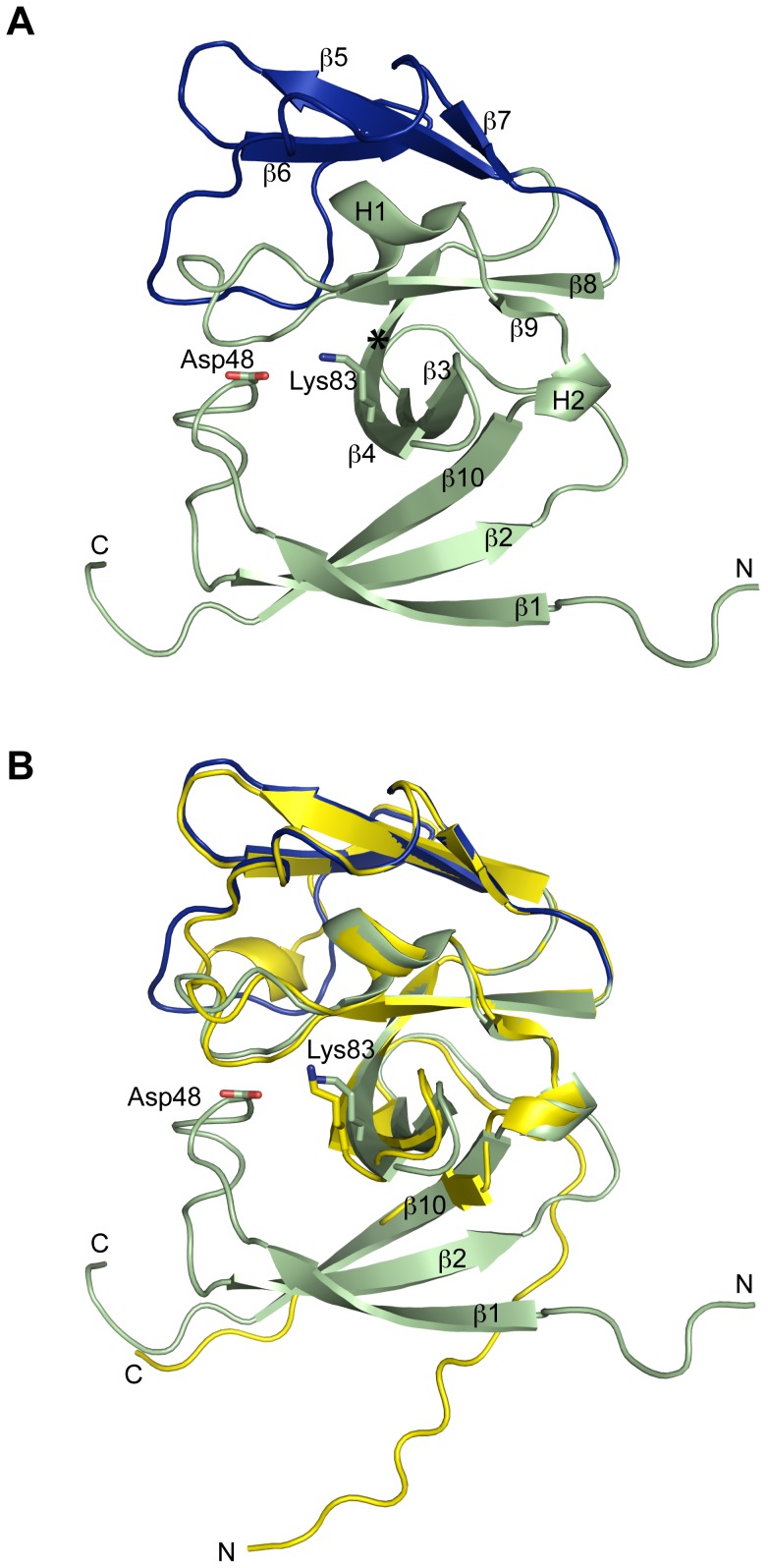
Ribbon diagrams of SipA. (**A**) The conserved catalytic core domain is shown in green and the non-catalytic 'cap' domain in blue. β-strands 1, 2 and 10 form a 'membrane associated' anti-parallel β-sheet that is preceded by a transmembrane helix, truncated in this construct. Residues potentially involved in catalytic activity, Asp 48 and Lys 83, are shown in stick form. Residues homologous to SPase-I catalytic dyad are Asp 48 and Gly 85. An asterisk shows the position of Gly 85 (*). (**B**) Structural superposition of SipA (green/blue) with SipA_trunc_ (yellow), a previously solved truncated structure, highlights the lack of the 'membrane associated' β-sheet (β-strands 1, 2 and 10) in the truncated structure. Neither the loop that positions Asp48 nor the peptide-binding cleft are present in SipA_trunc_. N = N-terminus, C = C-terminus.

The SipA structure was solved by molecular replacement using a previously solved truncated construct SipA_45-173_ [17] as a search model, and was then refined at 2.2 Å resolution (R = 20.1%, R_free_ = 22.9%; see *Table 1* for full details). The asymmetric unit contains two SipA monomers, A and B. For monomer A, interpretable electron density was obtained for the entire sequence, residues 36–173, together with four residues derived from the expression vector. The affinity tag could not be cleaved, and there is no electron density for the remaining 22 vector-derived residues, which include the His_6_ affinity tag and rTEV protease recognition sequence. Monomer B has no interpretable electron density prior to Val38, and has incomplete density for the loop between Arg63 and Arg67. The two monomers are almost identical except for some variations in the peptide-binding cleft, described later, with a root-mean-square-difference (rmsd) in Cα positions of 0.35 Å over 135 aligned residues. Unless otherwise stated, monomer A is taken as the representative model for SipA (Figure 1a).

**Table 1:** Data collection and refinement statistics.

Wavelength (Å)	0.95468	
No. of images	720	
Oscillation angle (°)	0.5	
*Resolution range (Å)	28.75–2.30 (2.42–2.30)	
*Total no. of observations	1086115 (159662)	
*Unique reflections	25314 (3618)	
*Redundancy	42.9 (44.1)	
Space group	*P*6_4_22	
Unit-cell axial lengths (Å)	*a* = 132.81, *b* = 132.81, *c* = 107.16	
angles (°)	*α* = 90, *β* = 90, *γ* = 120	
Molecules per A.U.	2	
Solvent content (%)	67.2	
*Completeness (%)	100 (100)	
Wilson Bfactor (Å^2^)	57.4	
*Mean *I/σ(I)*	32.2 (2.3)	
*R_merge_ (%) †	0.11 (2.28)	
*R_p.i.m._ (%) ◊	0.017 (0.35)	
*CC *Imean* §	1.0 (0.73)	
**Refinement statistics**		
*Resolution range (Å)	28.75–2.30 (2.42–2.30)	
R_work_ (%) ‡	19.7	
R_free_ (%) ‡	23.1	
Protein atoms	2158	
Water molecules	75	
R.m.s. deviation from ideal geometry		
Bonds (Å)	0.012	
Angles (°)	1.29	
Mean B-factor (Å^2^)	45.8	
Residues in the Ramachandran plot (MolProbity)		
Most favoured (%)	97.4	
Outliers (%)	0	

† R_merge_ = ∑_hkl_∑_i_ |I_i_ (hkl) -〈I(hkl)〉| /∑_hkl_∑_i_ I_i_ (hkl).

◊ R_p.i.m._ = ∑_hkl_ [1/(N-1)]^1/2^ ∑_i_ |I_i_ (hkl) -〈I(hkl) 〉| /∑_hkl_∑_i_ I_i_ (hkl).

‡ *R* = Σ_hkl_ | _|_F_o_(*hkl*)_|_ - _|_F_c_(*hkl*)_|_|/Σ_khl|_F_o_ (*hkl*)_|_. The *R* value is calculated using 95% of the data selected randomly and used in refinement. *R*_free_ is calculated from the remaining 5% of the data not used in refinement.

§ Mn(I) half-set correlation CC(1/2) as calculated by SCALA.

*Numbers in parentheses for outermost shell.

### SipA fold conforms to the Signal peptidase family

**Figure 2:**
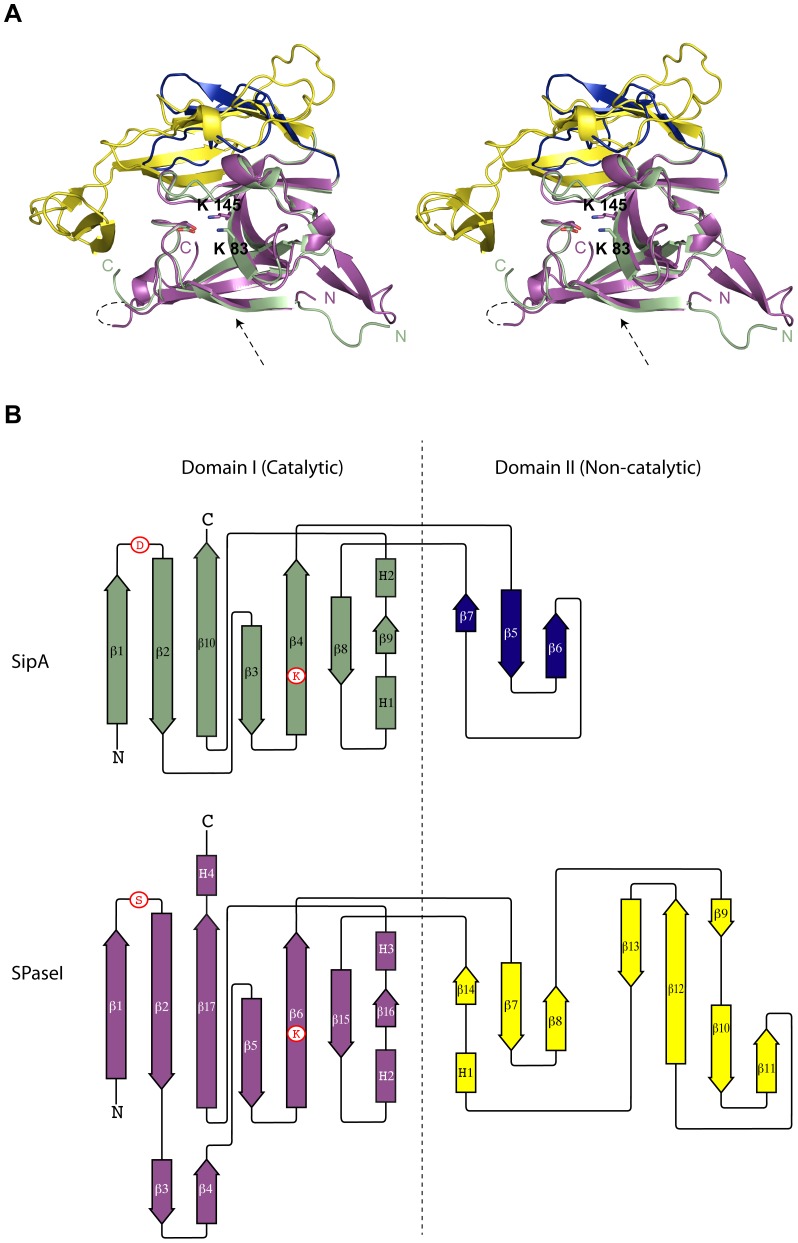
Comparison of SipA and *E. coli* SPase-I. (**A**) Stereo-view of a structural alignment between the extracellular domains of SipA and SPase-I. The conserved catalytic core domain of SipA and SPase-I is shown in green and magenta, respectively, and the non-catalytic 'cap' domains in blue (SipA) and yellow (SPase-I). Shown in stick form are the SPase-I catalytic dyad residues (Ser 90 and Lys 145) and the corresponding residues in SipA (Asp 48 and Gly 85, and the nearby Lys 83). An arrow depicts the position of the peptide binding clefts. (**B**) Topology diagrams of SipA and *E. coli* SPase-I, color-coded as for the ribbon diagram. Dashed lines represent regions not visible in the electron density. The positions of key catalytic residues are shown in circles. N = N-terminus, C = C-terminus.

Signal peptidases are a sub-family of the S24/S26 superfamily of serine peptidases that include the LexA repressors (S24) and type-I signal peptidases (S26). Members of this structural superfamily share a common catalytic domain (Domain I). The only available structure from the S26 signal peptidase family is that of *E. coli* SPaseI. SipA comprises two all-β domains that appear to be typical of the S26 signal peptidase family and is highly similar to *E. coli* SPaseI (Figure 2a). SipA domain I, the larger domain (residues 36-92 and 132–173), contains all the conserved sequence motifs of the SPase-I family (Boxes B–E), and is termed the ‘catalytic’ domain [29]. Domain II varies in size in different SPase family members, has no conserved sequence motifs, and is entirely missing in the LexA repressor subfamily [30], [31].

The present structure (Figure 1a), comprising the entire extracellular domain of SipA, differs in two very important respects from the previously reported truncated structure (Figure 1b). In the latter, the nine deleted N-terminal residues included the first β-strand of the catalytic domain. As a result, the 3-stranded β-sheet that in *E. coli* SPase-I is predicted to associate with the cell membrane was not present; instead strand β2 and the C-terminal strand β10 project out as an extended arm that mediates formation of an arm-swapped dimer. Neither the loop that provides the catalytic serine of SPase-I nor the peptide-binding cleft was formed.

In the present structure the 3-stranded sheet comprising strands β1, β2 and β10 is fully formed and presents an outer face populated with exposed hydrophobic residues (Tyr37, Phe39, Val41, Ile43, Leu60, Tyr62 Leu168 and Val170). Since strand β1 would be preceded by the N-terminal transmembrane α-helix, deleted in this construct, this sheet is predicted to associate with the cell membrane, as is proposed for *E. coli* SPase-I [19], [29]. The extended β1- β2 loop, which in *E. coli* SPase-I presents the catalytic serine residue in the active site, is also well-ordered and fills the same position, and the peptide-binding cleft, described below is also fully formed (Figure 2a). The result is that the SipA structure matches the SPase-I fold closely (Figure 2b). The ‘catalytic’ domain can be superimposed onto that of SPase-I with an rmsd of 1.25 Å for 93 equivalent residues, and for the whole molecule 127 residues can be superimposed with an rmsd of 1.67 Å. The main difference is in the much smaller non-catalytic domain of SipA, which is minimally decorated and similar in size to those of Gram-positive signal peptidases.

### SipA quaternary structure

Crystals of SipA were grown from a multimeric form predicted to be approximately 150 kDa, as determined by size exclusion chromatography and dynamic light scattering (DLS). Examination of the packing reveals that SipA forms an octamer whose basic unit is a dimer formed by molecules A and B, the asymmetric unit of the crystal. These associate through the antiparallel packing of their C-terminal β10 strands, forming an extended sheet. Two such dimers then associate through interaction of the N-terminal strand of molecule A with the equivalent strand of a neighbouring dimer related by crystal symmetry. This tetramer forms a horseshoe structure, with two such tetramers associating, again by crystal symmetry, to form the octamer (Figure S1).

The octamer appears to be significantly stabilized by unidentified molecules at the interface between the tetramers. The electron density is indicative of phospholipids, with a phosphate head-group and two lipid acyl chains, which we have modelled as phosphatidylethanolamine (PE). Four phospholipid molecules, presumed to originate from the *E. coli* host strain during purification, pack together at the interface between the tetramers. The head-groups make hydrogen bond contacts between molecules A and B from each tetramer, while the lipid acyl chains further stabilize the octamer with non-bonded contacts between residues from each tetramer (Figure S1). There is clear electron density for only one of the two acyl-chains of PE. An ill-defined acyl-chain sits close to an axis of symmetry and has been truncated to fit the interpretable density.

Small angle X-ray scattering (SAXS) analysis was used to determine whether the octamer present in the crystal structure is similar to that in solution, or whether the propensity for SipA to multimerise leads to other more biologically relevant complexes. Scattering data for SipA were collected across a range of concentrations at the Australian Synchrotron SAXS/WAXS beamline, and were analysed as described in the Supplementary Data (Table S2). The radius of gyration (Rg), as determined by Guinier analysis, was 36.88±0.20 Å, in close agreement with the value of 36.97±0.05 Å obtained from distance distribution analysis, calculated with GNOM [32]. The maximum dimension of the scattering particle (Dmax) was ∼112 Å, in agreement with the crystal structure of ∼105 Å (Figure S3).

**Figure 3:**
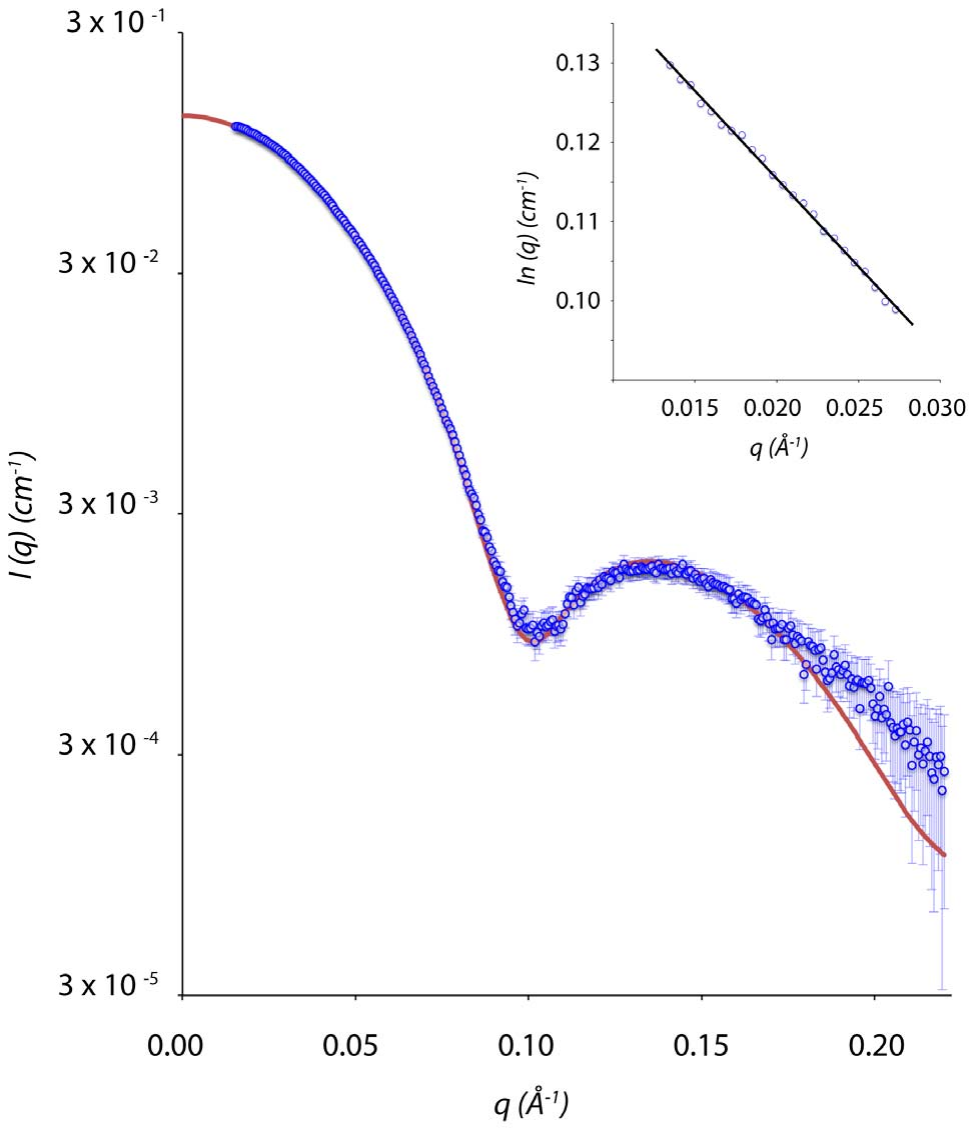
SAXS analyses of SipA. Superposition of the theoretical coordinate-derived scattering profiles from octameric SipA (solid line) and the raw SAXS data (○). Theoretical scattering profiles were generated from the SipA octamer crystallographic coordinates using CRYSOL. Inset: Guinier plot.

The theoretical scattering curve for the SipA octamer was computed using the crystal structure depicted in *Figure S1* with the program CRYSOL [33]. Superposition of the experimental and coordinate-derived scattering curves shows excellent agreement with χ^2^ = 1.29 (Figure 3). Low resolution data (∼500–40 Å, or q(Å-1)≤0.15) matches very well with the theoretical scattering calculated from the crystal structure. This suggests that the size and shape of the solution structure is equivalent to the crystal structure. The deviation between the experimental and calculated scattering in the medium angle scattering (q(Å-1)≥0.15) could represent domain movement, or reflect differences due to the 22 (x8) residues from the affinity tag that were not modelled in the crystal structure. Taken together, these data show that recombinant SipA adopts a multimeric conformation in solution very similar to the octameric structure observed in the crystal structure.

### Catalytic residues typical of signal peptidases are missing in SipA

The present structure shows that although SipA shares the SPase-I fold, the catalytic serine and lysine residues characteristic of active signal peptidases are missing, as was earlier suggested by sequence alignments [15], [16]. In SPase-I, Ser 90 acts as the essential nucleophile, with Lys 145 positioned to act as a general base [19], [34], [35]. The hydrophobic environment surrounding Lys 145 is thought to facilitate the lowering of its p*K*_a_ so that it exists in a deprotonated state necessary for both the acylation and deacylation steps of catalysis [19]. In contrast, SipA has an aspartic acid residue (Asp48) in the position of the SPase-I Ser 90, and a glycine (Gly85) at the site of SPase-I Lys145. Although SipA does possess a lysine residue only two residues removed from this site, Lys83, it is solvent exposed and seems unlikely to be able to act as a general base (Figure 2). Other differences include an invariant glycine (Gly272) in SPase-I, which is replaced by Asn140 in SipA. In SPase-I, Gly272 is adjacent to Lys 145, and any side chain at this position would clash with the catalytic Lys 145. This restriction is alleviated in SipA, which lacks the catalytic lysine. SPase-I Ser 278, involved in stabilization of Lys 145, is Arg 145 in SipA and points away from the ‘active site’. Consequently, SipA fails to show any detectable peptidase activity, as shown by the fact that when the precursor form of the major pilin (pre-FctA) is incubated with SipA, no processing is evident (data not shown). Similar conclusions were reached by Nakata *et al*. [16] in tests against pre-Cpa and synthetic peptides.

### Peptide-binding cleft

**Figure 4:**
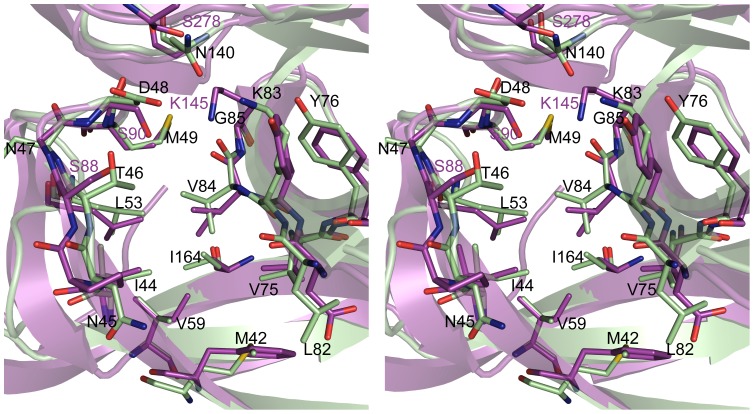
Residues lining SipA and *E. coli* SPase-I substrate binding pockets. Stereo-view of residues lining the substrate-binding pocket of SipA (green) and SPase-I (magenta). SipA residues (one letter code) are labeled in black text, with the SPase-I catalytic residues labeled in magenta. The pockets have the same orientation as *Figures 1* *and* *2*.

Although SipA lacks the catalytic residues of SPase-I, it retains the peptide-binding cleft found in *E. coli* SPase-I (Figure 2). This cleft, which is formed by residues from strands β1, β2, β5 and β6, has a high degree of similarity between the two proteins. The Cα positions in these strands overlay those of SPase-I with an rmsd of only 0.54 Å over 39 aligned residues, and the side chains that line the peptide-binding cleft are well conserved between the two proteins (Figure 4).

**Figure 5:**
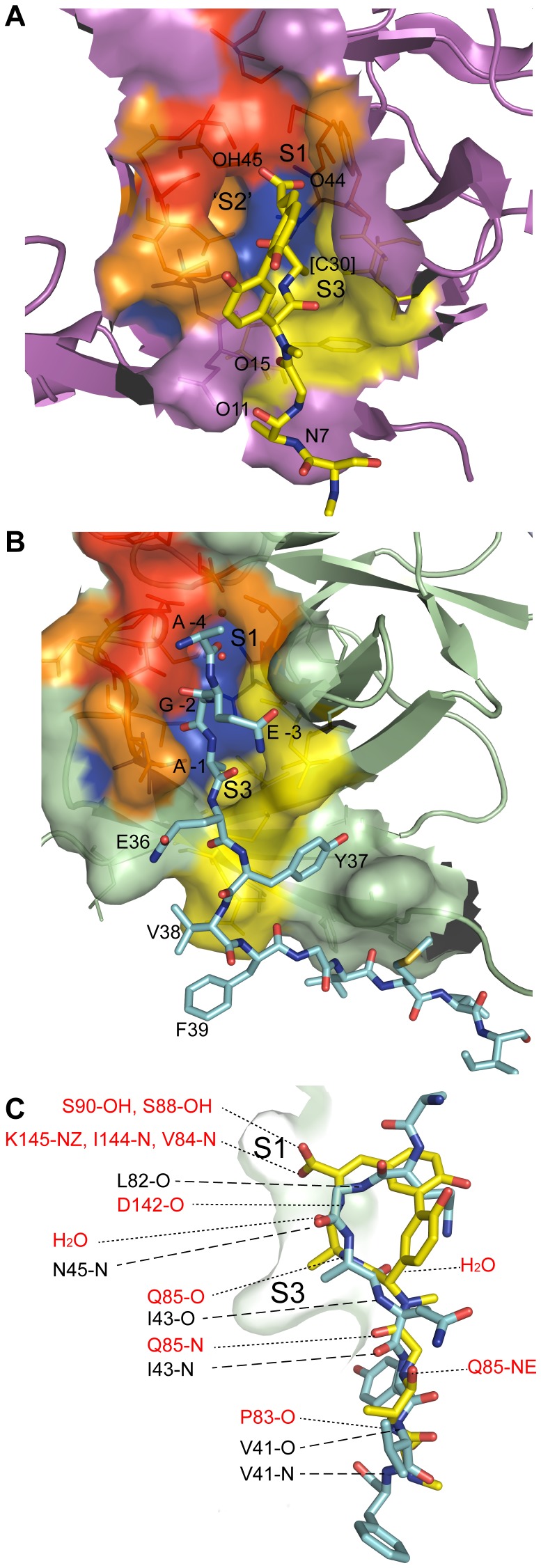
Comparison of the SipA and SPase-I substrate binding pockets. Surface representation of the substrate binding pockets of (**A**) *E. coli* SPase-I (PDB ID, 3IIQ) and (**B**) SipA. The molecular surface is colored red for residues involved in the catalytic center of SPase-I and the corresponding residues in SipA; orange for residues contributing side chain atoms to the S1 and S2 pocket; yellow for those residues contributing side chain atoms to the S3 pocket; and purple for residues bridging the two pockets. The SipA A' peptide (cyan) and arylomycin (yellow) are shown in stick form bound to SipA and SPase-I, respectively. (**C**) Superposition of the active sites of SipA and SPase-I showing hydrogen bond interactions. SipA residues are listed in black with large dashes, and SPase-I residues are in red with small dashes. Homologous residues are grouped. A' peptide (Gly -2 to Phe 39, cyan) and arylomycin (fatty acid tail not included, yellow) are shown in stick form as a side view in the substrate binding pocket, colored by element (carbon, cyan or yellow; oxygen, red; nitrogen, blue). A surface representation of the SipA pocket showing the S1 and S3 pockets is in green.

Analysis of the *E. coli* SPase-I structure identified two shallow hydrophobic pockets in the floor of the cleft, designated the S1 and S3 substrate-binding sites, predicted to accommodate the P1 and P3 residues (Ala-X-Ala) of signal-peptides [19], [36], [37]. A third pocket, designated the S2 sub-site and proposed to accommodate the P2 side chain [37], abuts the S1 pocket and forms the deepest cavity in the substrate binding cleft (Figure 5a). SipA contains hydrophobic pockets similar to the S1 and S3 pockets in SPase-I, but appears to lack an S2 pocket due to the rotamers adopted by the side chains of Thr46 and Val 84 (Figure 5b). Movement of these two side chains would, however, open up an S2 sub-site equivalent to that in SPase-I. At the head of the cleft, adjacent to the S1 site, the changes at the ‘catalytic’ site generate a polar pocket in SipA bounded by Asp48, Lys83 and Asn140, which provide a binding site for several water molecules.

The peptide-binding cleft extending from the S1 pocket to S3 has a volume of ca. 225 Å^3^ in SipA molecule A, or 270 Å^3^ if the S2 sub site is opened by altering the Thr46 and Val84 side chain rotamers. This compares with 300 Å^3^ for the SPase-I peptide-binding cleft (Q-sitefinder). In contrast, the peptide-binding cleft in SipA molecule B is smaller, at ca. 99 Å^3^, due to small rearrangements of side chains in the cleft. The side chains of Met42 and Asn45 move to occlude the S3 binding pocket, whereas Thr46 and Val84 adopt positions that open up sub site S2. This makes the point that the cleft is shallow but has some flexibility.

An intriguing feature of the SipA crystal structure is that the peptide-binding cleft of molecule A binds the N-terminal peptide of a symmetry-related molecule within the SipA octamer (Figure 5b). This N-terminal peptide (peptide A') is well ordered (Figure S2), with the three N-terminal residues Gln-Gly-Ala (residues -3 to -1, from the expression vector) positioned in the substrate-binding pocket. The methyl group of Ala-1' occupies the S3 pocket and Gly-2' makes nonpolar interactions with Thr46 and Val84, and main chain hydrogen bonds with Leu82 O and Asn45 N. These interactions induce a bend in the peptide chain such that Gln-3' is rotated away from the S1 pocket. Approximately 750 Å^2^ of solvent accessible surface area on SipA is buried by the binding of this N-terminal peptide. Interestingly, although this peptide binds in an orientation antiparallel to that expected for a signal-peptide, its binding closely resembles that of the lipohexapeptide arylomycin A2 to SPase-I [38] (Figure 5a). Homologous residues are involved in the interactions and the side chain methyl group of residue Ala-1' is positioned in the SipA S3 substrate pocket just as the C30 methyl group of arylomycin does in its binding to SPase-I (Figure 5c).

### Pilus polymerisation assays

At the amino acid sequence level SipA is highly conserved with almost 100% identity within all strains carrying the FCT types 3 and 4, but is more divergent in FCT2 strains with 44% identity (Figure S4a). The SipA protein characterised here was from a strain of *S. pyogenes* belonging to FCT3. As the requirement of SipA for pilus polymerisation had already been examined for a FCT3 strain [15], [16], we assessed the function of SipA from a more divergent FCT2 strain. To test its role in pilus polymerisation we expressed the complete pilus operon from the serotype M1/T1 strain SF370 (FCT2 type) in *Lactococcus lactis*. These included the genes for Cpa (Spy0125), SipA (Spy0127), FctA (Spy0128), SrtC1 (Spy0129) and FctB (Spy0130), which are conserved in number and gene order in all *S. pyogenes* strains that carry the *sipA* gene (FCT2, FCT3 and FCT4 type strains).

**Figure 6:**
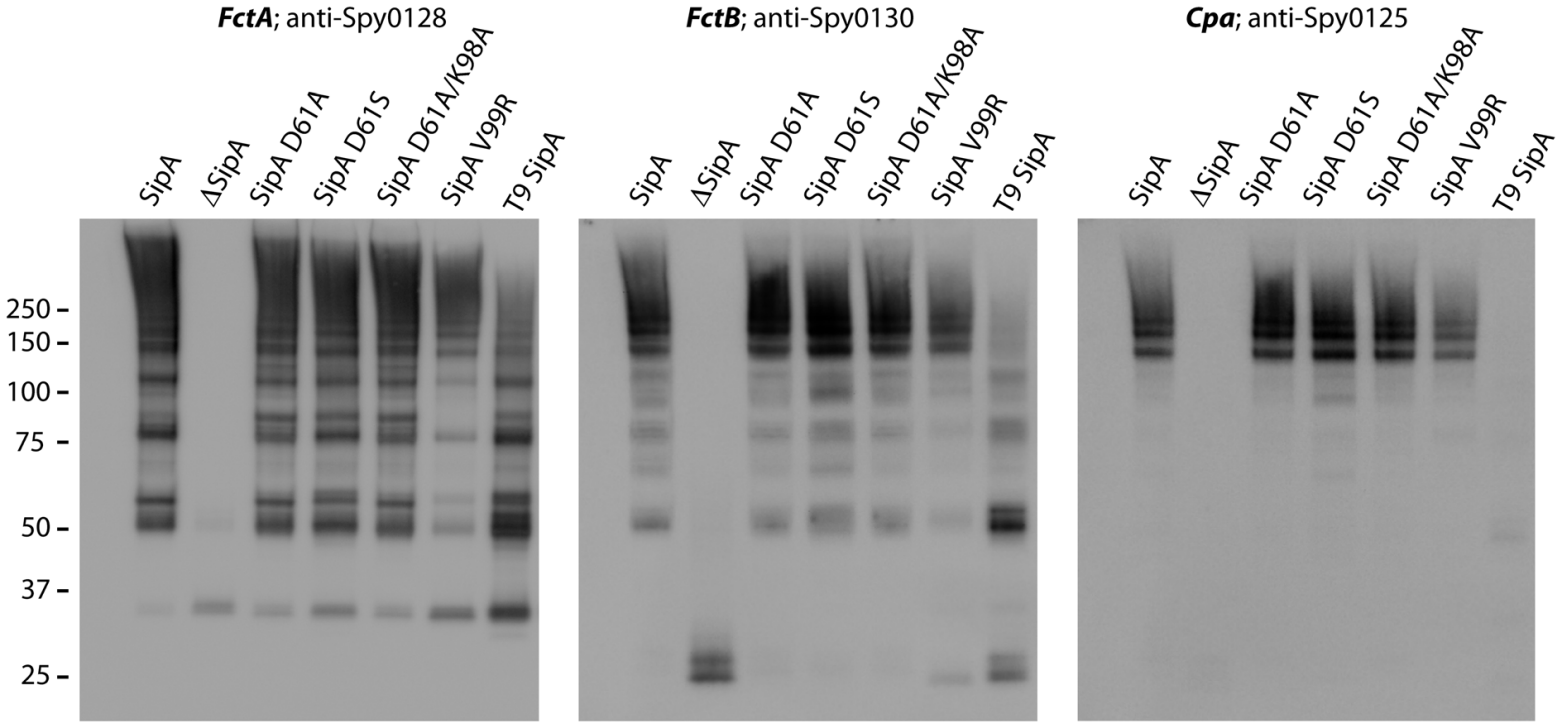
Western immunoblots of *L. lactis* expressing *S. pyogenes* pili. Cell wall extracts from *L. lactis* cells transformed with plasmids carrying the *S. pyogenes* M1/T1 strain pilus operon were immunoblotted with antisera against either FctA backbone pilin (anti-Spy0128), FctB minor pilin (anti-Spy0130), or Cpa adhesin (anti-Spy0125) to show the effect of SipA mutations on pilus polymerisation. Cells expressing WT levels of SipA show a laddering of high molecular weight polymers. SipA deletion mutant (ΔSipA) shows no pilin polymerisation, only monomeric FctA or FctB. Mutating aspartic acid D61 (SipA D61A and D61S) or the double mutation of SipA D61A/K98A, or the V99R S3 pocket-occluding mutant have minimal effect on pilus polymerisation, with each mutant producing high molecular weight polymers. Replacement of the M1/T1 SipA (FCT2) with T9 SipA (FCT3) produced pilus polymerisation, but with more lower molecular weight multimers including FctA-B dimers (∼50 kDa) and pili with no cpa. FctA = 32 kDa, FctB = 18 kDa, Cpa = 75 kDa.

Expression of the FCT2 pilus operon in *L. lactis* resulted in pilus polymerisation at the cell wall of *L. lactis* as indicated by high molecular weight polymers of the pilus backbone protein (Spy0128) protein in cell wall extracts, and the inclusion of both minor pilins into the pilus structure as indicated by detection of both Spy0130 and Spy0125 in the high molecular weight polymers in Western blots (Figure 6). Deletion of SipA resulted in the complete loss of pilus polymerisation, with only monomeric Spy0128 and Spy0130 subunits present in the cell wall (Figure 6); the majority of pilin proteins were located to either the cell membrane or cytoplasmic cell fractions (data not shown). This concurs with similar gene deletion studies in FCT3 strains [15], [16]. We similarly tested the roles of selected residues in SipA function. To evaluate the role of Asp61 in the M1/T1 SipA (equivalent to Asp48 in our T9 SipA structure or the nucleophilic serine in signal peptidases), D61A and D61S mutant versions of SipA were expressed in *L. lactis*. These mutants had no effect on pilus polymerisation indicating that this residue, essentially conserved in *S. pyogenes* SipA, plays no part in activity (Figure 6). A double mutant D61A/K98A (residues corresponding to T9 Asp48 and Lys83) also failed to affect pilus polymerisation (Figure 6). We also probed the effect of changes in the putative peptide-binding cleft by mutating Val99 (equivalent to Val84 in T9 SipA) to Arg. This was predicted to occlude the binding cleft and disrupt the hydrophobic S3 subsite by introducing a positive charge, but expression of this mutant in *L. lactis* did not effect polymerisation (Figure 6).

To test the redundancy of SipA function, a chimeric GAS serotype M1/T1 pilus operon with the T1 *sipA* (FCT2) substituted by T9 *sipA* (FCT3) was expressed in *L. lactis*. This chimeric operon still produced high molecular weight polymers of Spy0128, despite the use of the different SipA (44% identity with T1 SipA), suggesting that SipA is likely to have the same function in both serotypes of *S. pyogenes* (Figure 6). However, the efficiency of Spy0128 polymerisation was reduced and while Spy0130 was incorporated into the high molecular weight polymers there was a notable lack of the adhesin (Spy0125).

Finally, *in vitro* pulldown experiments were performed to test for possible interaction between SipA and the major pilus protein FctA. Truncated recombinant T9 FctA_(21-328)_ (pre-FctA), excluding the membrane spanning residues but retaining both the C-region of the signal-peptide and the C-terminal sortase motif, was mixed with N-terminally His-tagged T9 SipA_36-173_. Pulldowns with SipA failed to detect any association with recombinant pre-FctA. Experiments were repeated with both pre-FctA and T9 SrtC, but these also failed to show any interaction with SipA. Incubation of SipA with both SrtC and pre-FctA *in vitro* also failed to produce detectable polymers of FctA. Inclusion of detergent (1% TX-100) had no effect on any of the assay results.

### SipA peptide binding assays

To test whether SipA might function in binding peptides belonging to pilus components during assembly, peptides spanning the C-region of signal-peptides (including the first four residues from the mature protein) from serotype T9 Cpa, FctA and FctB, and the cleaved N-terminal region of the mature FctA protein were synthesized with a C-terminal fluorescein tag. The region surrounding the C-terminal sortase motif of FctA was also synthesized with an N-terminal fluorescein tag (Table 2). These peptides were each pre-incubated with purified SipA at either 18°C or 37°C, and pulldown experiments performed in an attempt to show an interaction between SipA and the peptides. Washing and elution steps were monitored at 480 nm, and showed no retention of any of the peptides with SipA. Finally, quantitative affinity chromatography was used to detect low affinity, weak molecular interactions between SipA and the peptides [39]. A small volume of peptide (25 µl of peptide at 10 µM, 50 µM, 100 µM and 1 mM concentrations) was injected on to a long thin IMAC column saturated with SipA (0.6 mM). Experiments over a range of peptide concentrations showed no difference in retention time in the column with or without SipA, for any of the peptides. These experiments suggest that recombinant SipA does not or cannot interact with peptides derived from pilin proteins, or that the interaction is too weak and transient to detect.

**Table 2:** Peptides used in SipA binding assays.

Protein	Peptide name	Sequence
Cpa signal-peptide	Cpa	FSIR**AFG**AEEQ(K) _FLUR_
FctA signal-peptide	FctA	MSQN**VKA**EGGV(K) _FLUR_
Mutant FctA signal-peptide	FctA_mut	MSQN**LKR**EGGV(K) _FLUR_
FctA N-terminal mature peptide	FctA_cut	EGGVSTGSILN(K) _FLUR_
FctB signal-peptide	FctB	FNQT**VLA**KDST(K) _FLUR_
FctA sorting peptide	FctA_QVPTG	_FLUR_ ***QVPTG***VVGTLAP

_FLUR_ = Carboxyfluorescein; ala-X-ala motif highlighted in bold text; sorting motif in bold italics.

## Discussion

The pili expressed by Gram-positive pathogens such as *S. pyogenes* are remarkable examples of covalent polymers whose assembly depends on the covalent linkage of successive pilin subunits in a process mediated by sortase transpeptidases. The pilin subunits are secreted *via* the Sec-dependent secretion pathway, and undergo processing at both their N-terminal and C-terminal ends as they are incorporated into the growing pilus. The N-terminal signal-peptide is removed, presumably by a housekeeping signal peptidase, and towards the C-terminus of the pilin a sorting motif (LPXTG or variant) is recognised and cleaved by a specific sortase. The latter then links the threonine carboxylate of the sorting motif *via* an isopeptide bond to the ε-amino group of a lysine residue in the next pilin subunit. Assembly thus depends on a series of peptide recognition events. Whether these events take place in the context of some larger complex is not known, but it is clear that assembly is absolutely dependent on the proper recognition and processing of specific peptide sequences.

How the signal peptidase homologue SipA participates in this process is unknown. Previous studies, and our own work described here, show that it is essential for polymerisation. SipA has previously been shown to be related to Type I signal peptidases but sequence comparisons have suggested that it lacks their typical Ser-Lys catalytic dyad, and must therefore either have some as yet unknown enzymatic activity [16] or have a chaperone-like function [15].

The present crystal structure shows unequivocally that SipA is homologous with *E. coli* SPase-I, with a very clear structural similarity, particularly in its catalytic domain (Figure 2). The two most striking features are that the catalytic apparatus of SPase-I is completely changed but that the peptide-binding cleft is strongly conserved. The catalytic residues Ser90 and Lys145, which sit at the end of the peptide-binding cleft in SPase-I, are replaced in SipA by Asp 48 and Gly 85. These residues occupy the same spatial position as the SPase-I catalytic residues and the loss of the catalytic Ser-Lys dyad is consistent with the loss of signal peptidase activity in SipA. Given its position, however, we sought to test whether Asp 48 has some alternative role in SipA function. We also noted that SipA has a conserved lysine (Lys 83) close to the position of the SPase-I catalytic lysine (Lys 145). The ε-amino group of Lys83 is only 4 Å from the side chain carboxylate of Asp48, and although it is fully solvent-exposed and unlikely to be able to act as a general base in the manner of Lys145, we considered the possibility that it could become buried upon complex formation with a substrate protein. Substrate-assisted catalysis has been known to occur in other systems [40], and there are a number of enzymes that use lysine-carboxylate pairs in acid-base chemistry [41].

To test the involvement of specific SipA residues in pilus polymerisation we used *L. lactis* as a surrogate host for expressing pili from *S. pyogenes* (M1/T1 strain SF370). This is an FCT2 strain and different from the one used for structural analysis of SipA, but with a sequence identity of 44% we can confidently predict they share the same fold, and the choice enabled us also to test the role of SipA in an FCT2 strain. We confirmed that FCT2 SipA is essential for polymerisation of both the major and minor pilins. Consistent with results from SipA deletion mutants in FCT3 pili [15], [16], deletion of SipA resulted in the complete loss of pilus polymerisation, with only monomeric backbone pilus subunits present in the cell wall. Mutating the Asp and Lys residues equivalent to Asp48 and Lys83 (Asp61 and Lys98 in FCT2 SipA) had no visible effect on pilin polymerisation. This indicates that these residues are not involved in any potential enzymatic activity. These residues, while largely conserved in *S. pyogenes*, are not conserved in SipA homologues from other species (Figure S4b) and we conclude that despite their position at the head of the conserved peptide-binding cleft they are not important for function.

Use of the *L. lactis* system also enabled us to show that substitution of FCT2 SipA by FCT3 SipA still leads to polymerisation of the major pilin, implying that SipA function is conserved across all strains of *S. pyogenes*. It is likely that SipA in other species also have the same function as those in GAS. However, while FCT3 SipA can substitute for FCT2 SipA in the polymerisation of FctA and the incorporation of FctB, it appears not to be able to assist in the incorporation of Cpa. This suggests that there is some sequence specificity.

Multiple sequence comparisons show that most conserved residues in SipA are likely to be retained because they are required for structural reasons. Notably, however, the putative peptide-binding cleft is almost entirely conserved, and is also highly conserved with respect to that in *E. coli* SPase-I (Figure 4). This suggests strongly that peptide binding is important in SipA function. Indeed, in our crystal structure the peptide-binding cleft of molecule A binds the N-terminal peptide chain from the adjacent SipA molecule. Although the strand orientation is anti-parallel, rather than parallel as in the proposed binding mode of signal-peptides to SPase-I, the peptide binding to SipA closely resembles that of arylomycin A2 to SPase-I (Figure 5) [38]. This suggests a common peptide binding function. It is also possible that the reverse orientation is functionally significant, possibly indicative of SipA binding to the C-terminal region of pilin proteins near the sorting motif; processing of a C-terminal peptide would very likely require such a reverse orientation.

In an effort to determine whether SipA interacts *in vitro* with either the signal-peptide or sortase motif from FctA (major pilin), we performed pull-down assays with the unprocessed major pilin pre-FctA, which retains the extracellular regions of both motifs (but lacking the cytosolic and membrane spanning sequences). We could show no association between FctA and SipA. Neither could we see any interaction between SipA and the pilus specific sortase, and no evidence of a SipA-sortase-FctA complex or the appearance of high molecular weight polymers of FctA. We also synthesised peptides encompassing the extracellular portion of signal-peptides of Cpa, FctA and FctB and the sorting motif region of FctA, but were unable to detect any interaction between any of these peptides and recombinant SipA.

Several factors militate against success in these binding experiments. Firstly, while each SipA monomer is thought to accurately represent the true physiological form, the octameric structure of our recombinant SipA (shown by SAXS to be present in solution as well as in the crystal) is unlikely to represent a physiological oligomer of SipA, which is expected to be membrane-associated. The presence of phospholipid in the interface between SipA molecules is suggestive of the orientation of SipA on the membrane, and is in agreement with the model for SPase-I. The octamer structure present in solution may inhibit the formation of biologically relevant complexes, for example with FctA. Half the peptide binding sites in the octamer are also occupied (by the N-terminal peptides of adjacent molecules) and although four remain free, binding to these sites could be sterically hindered by adjacent SipA molecules. We have so far been unable to obtain alternative soluble species. The complex cannot be dissociated with either non-ionic detergents or high ionic buffers. Secondly, studies with truncated signal peptidases have shown that their binding affinities are ∼100-fold lower than those of the wild type membrane-anchored enzyme, and that *in vitro* binding affinities are at least five-fold lower for synthetic peptides lacking both the N- (intracellular) and H- (transmembrane helix) regions than for pre-proteins [42]. If we assume that SipA can bind peptides, then we could also infer that their binding *in vitro* is likely to be weak, transient and difficult to measure.

Not all GAS pilus operons encode a *sipA*-like gene, so what is unique to the pili that require SipA for polymerisation? Pilus operons that encode SipA-like proteins are distinct in that they use a class-B sortase (spy0129) for polymerisation [43] whereas other Gram-positive organisms with similar pili, such as *S. pneumoniae*, *S. agalactiae* and *Corynebacterium diphtheriae* use class-C sortases [43], [44], [45]. There are also differences among the major pilins in terms of how the key lysine residue that participates in polymerisation is displayed. In most, such as SpaA from *C. diphtheriae*, this lysine is found in a YPKN pilin motif located on the last β-strand of the N-terminal domain [46]. In contrast, in the *S. pyogenes* major pilin Spy0128 the acceptor lysine is located on an omega loop near the top of the N-terminal domain [10]. The key lysine of the *S. pyogenes* basal pilin FctB is similarly positioned [13], and neither protein has the YPKN pilin motif. Is the structural context of the acceptor lysine a key factor in the requirement of both SipA and a class-B sortase for polymerisation, as opposed to a sole class-C sortase? Class-B sortases lack any equivalent to the flexible lid of the class-C enzymes, which is thought to be essential for recognition of pilin sorting motifs. In this context, SipA could function either in the direct recognition of pilin sorting signals, in concert with the class-B sortase, or form a scaffold that modifies and orientates the pilin proteins for optimal sortase transpeptidase activity.

Finally, recent evidence shows that even active signal peptidases can be involved in functions independent of their peptidase activity. The import of the antibacterial toxin colicin D into *E. coli* is dependent on SPase-I (also known as LepB) [47], but is independent of LepB catalytic activity. Instead it is proposed that LepB has a structural role, modifying the structure of a colicin D domain to allow proteolysis by the inner membrane protease FtsH [47]. This role of LepB as a scaffold to modify or hold colicin D in a specific conformation is analogous to the role we propose for SipA in pilus polymerisation. In this model SipA associates with the newly secreted pilin and holds it in a specific conformation, potentially partially unfolded, which allows for efficient pilin polymerisation by sortase. The exact nature of this interaction, however, remains unknown.

## Materials and Methods

### Strains and growth conditions

*Escherichia coli* DH5-alpha (Invitrogen) and BL21 (DE3) pRIL (Stratagene) were cultured at 37°C in LB media supplemented with the appropriate antibiotic (150 µg/ml chloramphenicol, 100 µg/ml ampicillin and 5 µg/ml erythromycin). *Lactococcus lactis* strain MG1363 was cultured without shaking at 28°C in M17 media (Gibco) supplemented with 0.5% glucose (GM17). When appropriate, 34 µg/ml of erythromycin was added. *Lactococcus lactis* was made competent by the method of Holo and Nes [48].

### Cloning, expression and purification of SipA

SipA was cloned and expressed as described previously [17]. Briefly, the *sipA* gene comprising the entire extracellular region of the protein, residues 36–173, (SipA_36-173_) was PCR-amplified from *S. pyogenes* serotype T9 strain 90/306S genomic DNA using the gene specific primers SPY0127 F1 and SPY0127 R1 (Table S1). The amplified fragments were cloned into the vector pProEXHTa (Invitrogen) or in pProEXHTa modified to contain a Maltose Binding Protein (MBP) between the N-terminal His-tag and the rTEV protease recognition site, and transformed into *E. coli* BL21 (λDE3) pRIL cells for recombinant protein expression. After IPTG induction, the cells were harvested and stored at −20°C as previously described [17]. Cells were thawed and then lysed using a cell disruptor (Constant Cell Disruption Systems) at 18 kpsi.

After centrifugation (30000 g, 4°C, 30 min) to remove insoluble matter, the recombinant SipA was purified by IMAC as previously described [17], but with the eluted protein collected into an equal volume of glycine buffer (25 mM Tris.Cl pH 8.0, 250 mM glycine). In a final step, SipA protein was concentrated and purified by size exclusion chromatography on a Superdex S200 10/300 column (GE Healthcare) in crystallization buffer (10 mM Tris.Cl pH 8.0, 100 mM NaCl). SipA eluted in a single peak and was approximately 99% pure as indicated by SDS-PAGE. Dynamic light scattering (DLS) data confirmed the protein was mono-dispersed with a radius of gyration that equates to a molecular weight of 150 kDa, consistent with the elution peak from size exclusion chromatography.

### Crystallization

Crystallization conditions were identified by sitting-drop vapour diffusion at 18°C, using 200 nl drops (100 nl each of protein and precipitant) dispensed by a Cartesian nanolitre dispensing robot (Genomic Systems), with a locally compiled crystallization screen [49]. Initial SipA crystals were subsequently optimised by hanging-drop vapour diffusion. The crystals used for X-ray data collection grew by mixing 1 µl protein solution (20 mg/ml in 10 mM Tris.Cl pH 8.0, 100 mM NaCl) with 1 µl precipitant (1 M NaKPO_4_ pH 7.0, 8% MPD) at 18°C.

### Data collection and structure determination

Crystals of SipA were transferred to cryoprotectant (1 M NaKPO_4_ pH 7.0, 8% MPD, 25 mM Tris.Cl pH 8.5, 10% (v/v) glycerol) prior to flash cooling in liquid nitrogen. X-ray diffraction data were recorded on a Quantum-315 CCD detector at the MX2 beamline of the Australian Synchrotron. All data sets were integrated using XDS [50], re-indexed using POINTLESS [51] and scaled using SCALA [51]. The crystals belong to the hexagonal space group *P*6_4_22. The unit cell dimensions were determined to be a = 132.8, b = 132.8, c = 107.2, and α = 90, β = 90 γ = 120. The solvent volume of the crystal was calculated to be 67.2%, with two molecules in the asymmetric unit. The resolution cut-off for the SipA (2.3 Å) was based on both I/σ(I) (empirical signal-to-noise ratio of ∼2.0) and CC 1/2 values as described by Karplus and Diederichs (2012) [52].

The structure of SipA_36-173_ was determined by molecular replacement with Phaser [53] using the previously solved truncated SipA structure, SipA_Δ9_ as the search model (PDB entry 4k8w, Young *et al*, 2013). The structure was then refined using iterative cycles of manual building in COOT [54], and refinement with REFMAC [55]. Model quality was monitored using PROCHECK [56]. Data collection and refinement statistics are shown in Table 1. All figures were generated using PyMOL (The PyMOL Molecular Graphics System, Version 1.5.0.4 Schrödinger, LLC). The coordinates and structure factors of SipA have been deposited in the Protein Data Bank under the accession code of 4N31.

### Small angle X-ray scattering analysis

Small Angle X-ray Scattering (SAXS) data were collected at the Australian Synchrotron SAXS/WAXS beamline equipped with a Pilatus detector (1 M, Dektris). The wavelength of the X-rays was 1.0332 Å. The sample detector distance was 3400 mm, providing an s range of 0.0007–0.0341 Å^−1^ (s is the magnitude of the scattering vector, related to the scattering angle (2θ) and wavelength (λ) by: s = (4π/λ) sinθ). Buffers/samples were loaded into 1.5 mm quartz capillaries and continuously flowed through the beam at a rate of 4 µl/sec during data collection to control radiation damage. SAXS measurements are the average of ten 1 s exposures. A dilution series of the protein samples was measured at concentrations between 1 and 20 mg/ml.

Background correction, averaging, and scaling were done with SAXS15ID software. Further processing was carried out using the ATSAS programme suite (version 2.4.3) (http://www.embl-hamburg.de/biosaxs/software.html). Data quality was assessed on the basis of the linearity of Guinier plots and *R*g, and the pairwise intraparticle distance distribution function (Pr) was determined using GNOM [57]. Theoretical scattering curves were generated from atomic coordinates and compared with experimental scattering curves using CRYSOL [33].

### Pulldown and peptide-binding assays

The DNA encoding pre-FctA_(21–328)_ and SrtC_(32–239)_ were amplified by PCR from *S. pyogenes* strain 90/306S genomic DNA, and the recombinant proteins were expressed in *E. coli* and purified as previously described [17]. Pre-FctA retains the entire extracellular portion of the protein including the signal peptidase (C-region) and sorting (LPXTG) motifs, while SrtC has both the signal peptide and C-terminal transmembrane domain truncated. For pulldown experiments, FctA (20 µg), SrtC (15 µg) and SipA (15 µg) were mixed to a total volume of 50 µl in 50 mM Tris.Cl pH 8.0 and 150 mM NaCl, with or without 5 mM β-mercaptoethanol, and incubated for 60 minutes at 37°C. A sample was taken as a control and the remaining volume passed through a His-SpinTrap column (GE Healthcare). The flow-through was collected, and the beads washed three times with buffer containing 20 mM imidazole. Bound proteins were eluted with 500 mM imidazole and analysed on 12% SDS-PAGE gels electrophoresis. Experiments were performed with either SipA or SrtC as the His-tagged target proteins, or with SipA expressed with a maltose binding protein affinity tag bound to amylose resin (New England Biolabs). Pulldown experiments were also performed in the presence of 1.0% TX-100.

Peptides encompassing either the extracellular region (C-region) of the signal-peptide or sorting signal region of pilin proteins from *S. pyogenes* strain 90/306S were synthesised using microwave Fmoc SPPS [58] and labelled with a fluorescein-tag as previously described [59] (Table 2). For pulldown assays, peptides were pre-incubated with purified SipA at either 37°C or 18°C. Washing and elution steps were monitored at 480 nm. For quantitative affinity chromatography, 25 µl of peptide at 10 µM, 50 µM, 100 µM and 1 mM concentration was injected on to an IMAC column (100 mm×2 mm, NTA) pre-saturated with SipA (0.6 mM). The retention time from each of the peptides was monitored at both 280 and 480 nm, with and without pre-bound SipA.

### Polymerisation and peptidase assays

Pre-FctA (20 µg), SrtC (15 µg) and SipA (15 µg) in 50 mM Tris.Cl pH 8.0 and 150 mM NaCl were mixed with or without 5 mM β-mercaptoethanol and 1% TX-100 to a total volume of 50 µl and incubated for 20 h at 37°C. The reactions were analysed on 12% SDS-PAGE gels electrophoresis, and examined for evidence of FctA polymerisation with silver-staining. For peptidase assays, pre-FctA (20 µg) and SipA (15 µg) were mixed with or without 1% TX-100 and incubated for 20 h at 37°C, and analysed for cleavage of pre-FctA.

### Expression of *S. pyogenes* FCT2 pilus operon in *L. lactis*

The construct pOri23:PilM1WTSipA, encompassing the FCT2 pilus operon genes *spy0125* to *spy0130* from GAS strain M1 SF370 (assembly ASM678v1), and a modified *sipA* deletion mutant (pOri23:PilM1Δ*sipA*) were produced as described below. To delete *sipA*, the pilus operon was amplified using gene-specific primers in two separate rounds of PCR amplification encompassing first *spy0125 (cpa)*, and then *spy0128* to *spy0130*. *Spy0125* was amplified using the PCR primers PilM1 BamHI F and M1SipA del R, and the *spy0128-spy0130* fragment with primers M1SipA del F and PilM1 SalI R2 (Table S1). As the reading frames for *spy0125* and *sipA (spy0127)* overlap by eight base pairs a XhoI restriction endonuclease site was introduced into the DNA region that encodes the intracellular region of SipA. By manipulating codon usage the translated sequence was left unchanged. A stop codon was introduced after the XhoI site (M1SipA del F primer). As a result the Δ*sipA* construct expresses the first 12 amino acids of the intracellular portion of SipA. The *spy0125* and *spy128-spy130* fragments were sub-cloned into a modified pBluescript II-KS vector with a MCS containing sequential BamHI, XhoI, KasI restriction endonuclease sites to generate the Δ*sipA* construct, which was sequence verified. The PilM1-Δ*sipA* expression construct was produced by excising the BamHI-SalI fragment and cloning into the pOri23 plasmid [60]. As a positive control for the deletion construct, *sipA* was re-cloned into pOri23:PilM1-Δ*sipA* to produce pOri23:PilM1WT*sipA*. *SipA* was PCR amplified using the gene specific primers PilM1 SipA F and PilM1 SipA R (Table S1). The resulting PCR product was digested with XhoI and NotI and cloned into pOri23:PilM1Δ*sipA* digested with XhoI and NotI, which removes the Δ*sipA* stop codon. The final constructs pOri23:PilM1WT*sipA* and pOri23:PilM1Δ*sipA* retain the native ribosomal binding sites for each of the genes in the operon, with the only additional non-native sequence a NotI restriction endonuclease site introduced into the noncoding region between *sipA* and *spy0128*. The PilM1-T9*sipA* chimeric operon was constructed by amplification of T9 *sipA* from *S. pyogenes* strain 90/306S genomic DNA using the gene specific primers T9SipA F1 and T9SipA R1. The resulting PCR product was digested with XhoI and NotI and cloned into pOri23:PilM1Δ*sipA* as described for WT M1*sipA*. All constructs were sequence verified.

### Mutagenesis

The gene for M1/T1 SipA was PCR amplified using gene-specific primers PilM1 SipA F and PilM1 SipA R (Table S1) and subcloned into pBluescript II-KS vector. Inverse PCR site-directed mutagenesis was used to modify selected residues [61]. Briefly, a high fidelity DNA polymerase (pfu Ultra II fusion HS, Stratagene) was used for the PCR amplification of the pBluescript:*sipA* construct to produce a linearized PCR product with the desired mutation at the 5' end of the sense primer. Template vector was removed by DpnI digestion, which digests only methylated DNA, and then re-circularized by intra-molecular ligation to produce a modified construct. Primers used for mutagenesis are listed in Table S1. Mutants were sequence-verified and cloned into pOri23:PilM1Δ*sipA* digested with XhoI and NotI.

### Preparation of *L. lactis* cell wall fractions

Overnight cultures of *L. lactis* strain MG1363 transformed with either pOri23:PilM1Δ*sipA* or pOri23:PilM1WTSipA were washed once and concentrated 10-fold in saline. Cell wall extraction was performed using 4 ml of cells (optical density at 600 nm of 2.0) in lysis buffer (50 mM Tris.Cl pH 6.8, 30% raffinose, 4 mg/ml lysozyme (Sigma-Aldrich), 400 U/ml mutanolysin (Sigma-Aldrich), Roche complete protease inhibitors) at 37°C for 3 h with constant rotation [62]. Cell debris was pelleted and the supernatant fraction containing the cell wall fraction was collected. SDS PAGE and Western blot analysis were performed and pilus formation was monitored by the appearance of high-molecular-weight (HMW) bands in immunoblots using antisera against Spy0125, Spy0128, or Spy0130.

## Supporting Information

Figure S1 **Ribbon diagrams of SipA quaternary structure.** (**A**) The asymmetric unit of SipA is a dimer of molecule A1 and B1. The conserved catalytic core domain is shown in green and the 'non-catalytic' cap domain light blue. Phosphatidylethanolamine (PE) molecules are shown in stick form. (**B**) A horseshoe shaped tetramer consists of two dimers (A1-B1 and A2-B2) related by 2-fold symmetry, with the N-terminal portion of β-strand 1 of each molecule-A occupying the peptide-binding cleft in the adjacent molecule-A. (**C**) An octamer consists of two horseshoe-like tetramers that interlock. Four PE molecules buried in the center of the complex. The arrow highlights the N-terminal peptide of A3 (yellow) bound in the peptide-binding groove of A4 (blue). N = N-terminus, C = C-terminus.
(TIF)

Figure S2 (**A**) Scattering data for SipA collected across a range of concentrations (1–20 mg/ml). Concentrations at 5 mg/ml (blue) and 20 mg/ml (yellow) show evidence of interparticle interference, which is characteristic of a downturn in the scattering plot. (**B**) Guinier plots of SAXS data of SipA at 1.25 mg/ml (a) and 2.5 mg/ml (b). (**C**) P(r) function calculated from SipA SAXS data. The experimental P(r) function was calculated using GNOM (3) and error bars indicate uncertainty in P(r) propagated from I(s) versus *s* profile.
(TIF)

Figure S3 **Ribbon diagram of SipA showing peptide A' bound in the peptide binding cleft.** The peptide is in stick form, colored by atom type, in electron density from a 2Fo - Fc map contoured at 0.21 eÅ^−3^ (1.3σ).
(TIF)

Figure S4 (**A**) Pairwise sequence alignment of the extracellular domains of T9 and T1 SipA (**B**). Sequence alignment of SipA homologues in *Streptococcus* species with key residues highlighted.
(DOC)

Table S1 **PCR Primer details.**
(DOC)

Table S2 **SAX data-collection and scattering-derived parameters.**
(DOC)
