# Supplementary figures and images for: Structure and Activity of Streptococcus pyogenes SipA: A Signal Peptidase-Like Protein Essential for Pilus Polymerisation
Source: PLoS One. 2014 Jun 9;9(6):e99135. doi: 10.1371/journal.pone.0099135 (PMC4049620; doi:10.1371/journal.pone.0099135)

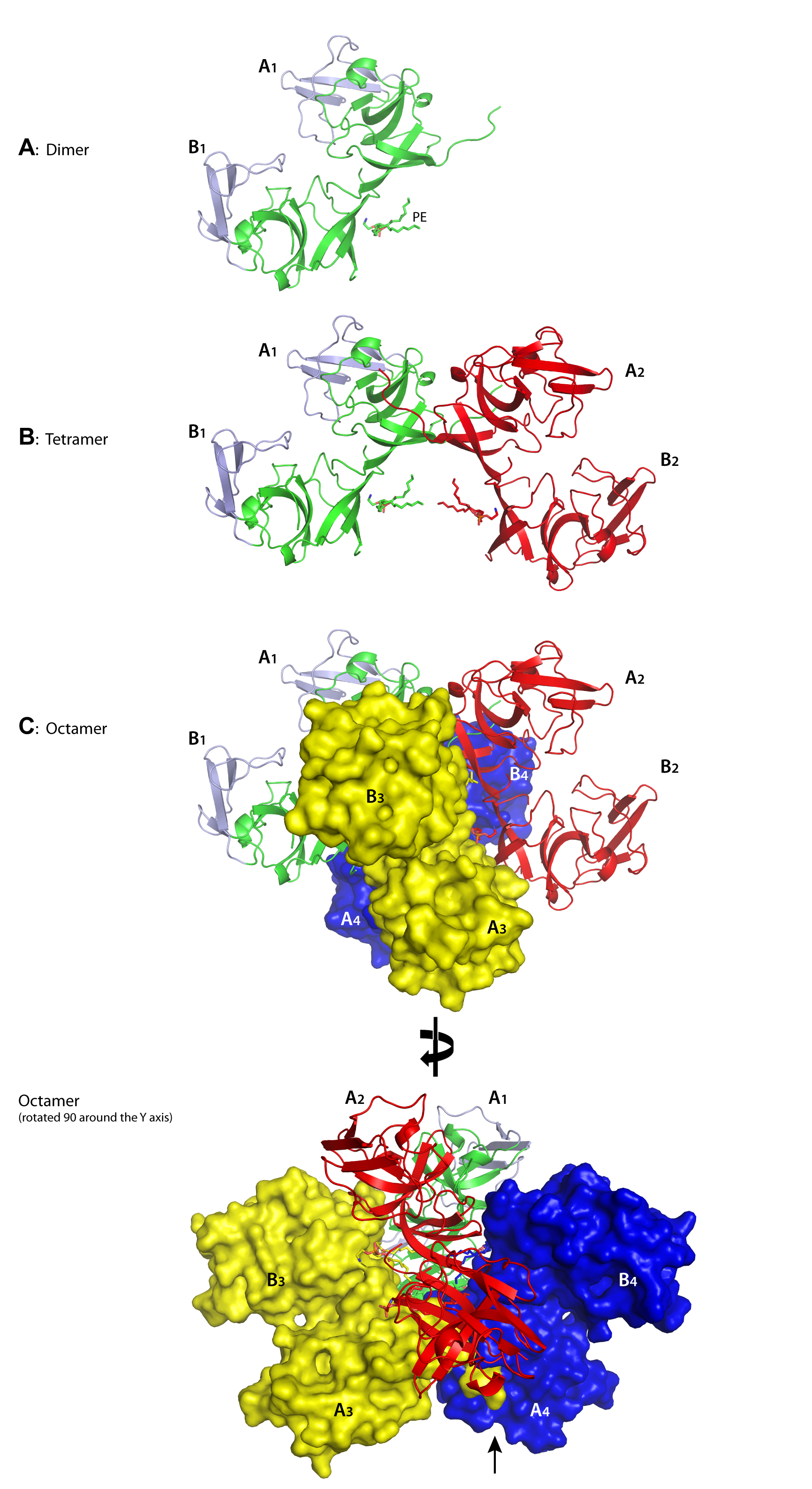

Supplement: Figure S1 — Ribbon diagrams of SipA quaternary structure. (A) The asymmetric unit of SipA is a dimer of molecule A1 and B1. The conserved catalytic core domain is shown in green and the 'non-catalytic' cap domain light blue. Phosphatidylethanolamine (PE) molecules are shown in stick form. (B) A horseshoe shaped tetramer consists of two dimers (A1-B1 and A2-B2) related by 2-fold symmetry, with the N-terminal portion of β-strand 1 of each molecule-A occupying the peptide-binding cleft in the adjacent molecule-A. (C) An octamer consists of two horseshoe-like tetramers that interlock. Four PE molecules buried in the center of the complex. The arrow highlights the N-terminal peptide of A3 (yellow) bound in the peptide-binding groove of A4 (blue). N = N-terminus, C = C-terminus. (TIF) [file pone.0099135.s001.tif]

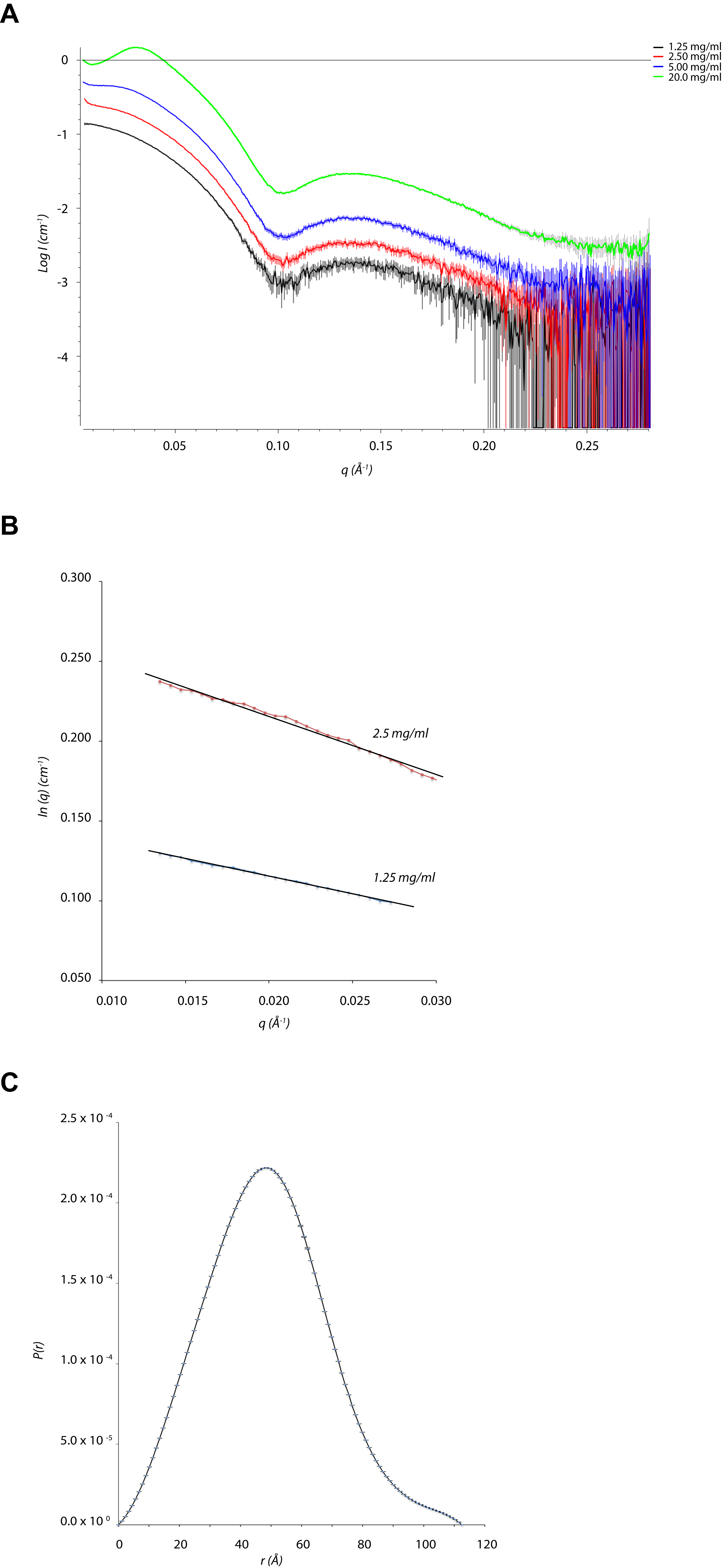

Supplement: Figure S2 — (A) Scattering data for SipA collected across a range of concentrations (1–20 mg/ml). Concentrations at 5 mg/ml (blue) and 20 mg/ml (yellow) show evidence of interparticle interference, which is characteristic of a downturn in the scattering plot. (B) Guinier plots of SAXS data of SipA at 1.25 mg/ml (a) and 2.5 mg/ml (b). (C) P(r) function calculated from SipA SAXS data. The experimental P(r) function was calculated using GNOM (3) and error bars indicate uncertainty in P(r) propagated from I(s) versus s profile. (TIF) [file pone.0099135.s002.tif]

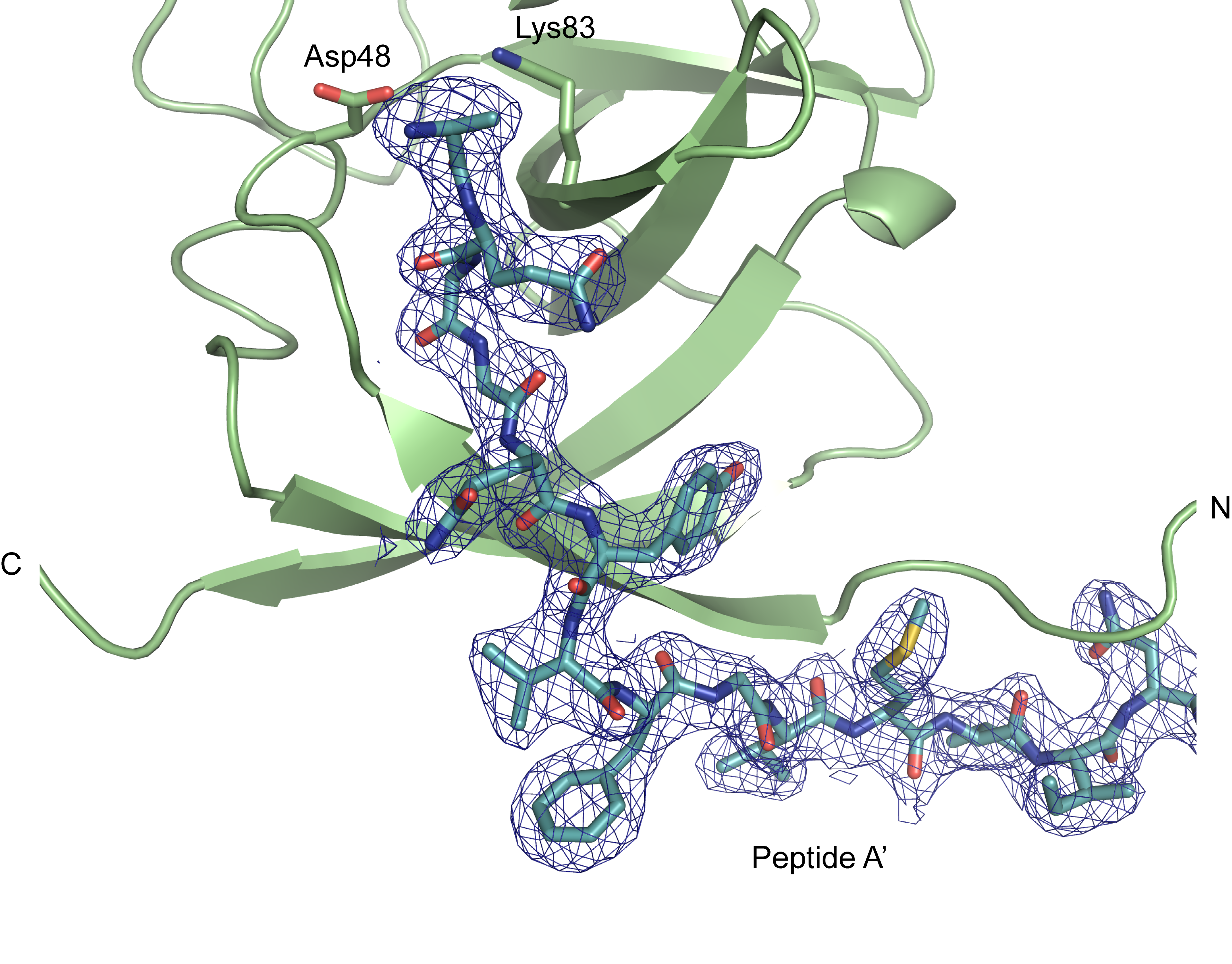

Supplement: Figure S3 — Ribbon diagram of SipA showing peptide A' bound in the peptide binding cleft. The peptide is in stick form, colored by atom type, in electron density from a 2Fo - Fc map contoured at 0.21 eÅ−3 (1.3σ). (TIF) [file pone.0099135.s003.tif]
