# Supplementary material for: Structure and Activity of Streptococcus pyogenes SipA: A Signal Peptidase-Like Protein Essential for Pilus Polymerisation
Source: PLoS One. 2014 Jun 9;9(6):e99135. doi: 10.1371/journal.pone.0099135 (PMC4049620; doi:10.1371/journal.pone.0099135)
Supplement: Figure S4 — (A) Pairwise sequence alignment of the extracellular domains of T9 and T1 SipA (B). Sequence alignment of SipA homologues in Streptococcus species with key residues highlighted. (DOC) [file pone.0099135.s004.doc]

**Figure S4**

**A:** Pairwise alignment of T9 SipA vs M1/T1 SipA

T9 QYVFGVMIINTN**D**MSPALSAGDGVLYYRLTDRYHINDVVVYEVDNT--L**K**VGRIAAQAGDEVSFTQEGGLLINGHPPEKE

:::.:::. :::.:...:::..:.:::.. :.....:::: ..: ::::: :::::::..:..: : :::: ..

T1 HFVFGFMIIKRN**D**MAPSVKAGDAILFYRLSQTYKVEEAVVYEDSKTSIT**K**VGRIIAQAGDEVDLTEQGELKINGHIQNEG

T9 VPYLTYPHSSGPNFPYKVPTGTYFILNDYREERLDSRYYGALPINQIKGKISTLLRVRGI

::. .: :.::.. ..:.::::: .. .. :. . ::: :.::.:.:

T1 ---LTFIKSREANYPYRIADNSYLILNDYYSQESENYLQDAIAKDAIKGTINTLIRLRNH

**B:** 'Non-peptidase' SipA homologues in *Streptococcus* species.

Transmembrane

"catalytic residues"

Non-catalytic domain

conserved salt bridge in SPases

conserved glycine in active SPases

Normal position for base lysine

S. pyogenes T9 -------------MTNYLNRLNENSLFKAFIRLVLKISIIGFLGYILFQY 37

S. pyogenes M5 -------------MTNYLNRLNENSLFKAFIRLVLKISIIGFLGYILFQY 37

Streptococcus_dysgalactiae -------------MTRYMNYLKNNILVNSIVRLMLKILVVILLAYLLFQY 37

SipA_Streptococcus_oralis_Uo5 ---MLLKKKHKKTVTQVNRDKSPPSVWGDILYLVSKLLMVGFVLATLYFF 47

LepB-Streptococcus-mitis-ATCC6 ---MLLKKKYKKTVTQVNRDKSPPSVWGDILYLVSKLLMVGFVLATLYFF 47

LepB_Streptococcus_sanguinis_A ---MLLKKKHKKTVTQVNRDKSPPSVWGDILYLVSKFLMVGFVLATLYFF 47

Streptococcus_pneumoniae ---MLLKKKHKKPVTQVNRDKSPPSVWGDILYLVSKLLMVGFVLAILYFF 47

S. pyogenes M1 MTRADFRTSNSSGELNKLSFSDKEAIKPLLIGLVKKIALILIGGYLLFHF 50

. . : :: *: *: :: : *: :

S. pyogenes T9 VFGVMIINTNDMSPALSAGDGVLYYRLTDRYHINDVVVYEVDNT--LKVG 85

S. pyogenes M5 VFGVMIINTNDMSPALSAGDGVLYYRLTDRYHINDVVVYEVDNT--LKVG 85

Streptococcus_dysgalactiae VFGLMIVKTNHMSPAINAGDGVLYYRLTDRYHINDVVVYEIDNT--LKVG 85

SipA_Streptococcus_oralis_Uo5 VFGLLRYNDDGMKPALKDGDLVVYYRLDKRYSIGDLLVYSYKGK--ERVA 95

LepB-Streptococcus-mitis-ATCC6 VFGLLRYNDDGMKPALKDGDLVVYYRLDKRYSIGDLLVYSYKGK--ERVA 95

LepB_Streptococcus_sanguinis_A VFGLLRYNDDGMKPALKDGDLVVYYRLDKRYSIGDLLVYSYKGK--ERVA 95

Streptococcus_pneumoniae VFGLLRYNDDGMKPALKDGDLVVYYRLDKRYSIGDLLVYSYKGK--ERVA 95

S. pyogenes M1 VFGFMIIKRNDMAPSVKAGDAILFYRLSQTYKVEEAVVYEDSKTSITKVG 100

***.: : : * *::. ** :::*** . * : : :**. . . :*.

S. pyogenes T9 RIAAQAGDEVSFTQEGGLLINGHPP-EKEVPYLTYPHSSGPNFPYKVPTG 134

S. pyogenes M5 RIVAQAGDEVSFTQEGGLLINGHPP-EKEVPYLTYPHSSGPNFPYKVPTG 134

Streptococcus_dysgalactiae RIVAQGDDEVNFTEDGGLLVNGHPP-EKEVPYLTYPHSSGPNFPYKVPKN 134

SipA_Streptococcus_oralis_Uo5 RVIATEGSTIDIN-ENGLIINGSPQQEQDIYKETLLYKEGATFPMKVPAG 144

LepB-Streptococcus-mitis-ATCC6 RVIATEGSTIDIN-ENGLIINGSPQQEQDIYKETLLYKEGATFPMKVPAG 144

LepB_Streptococcus_sanguinis_A RVIATEGSTIDIN-ENGLIINGSPQQEQDIYKDTLLYKEGASFPMKVPAG 144

Streptococcus_pneumoniae RVIATEGSTIDIN-ENGLIINGSPQQEQDIYKETLLYKEGATFPMKVPAG 144

S. pyogenes M1 RIIAQAGDEVDLTEQGELKINGHIQ----NEGLTFIKSREANYPYRIADN 146

*: * .. :.:. :. * :** * . ..:* ::. .

S. pyogenes T9 TYFILNDYREERLDSRYYGALPINQIKGKISTLLRVRGI 173

S. pyogenes M5 KYFILNDYREERLDSRYYGALPVNQIKGKISTLLRVRGI 173

Streptococcus_dysgalactiae TYFILNDYREERLDSRYYGALPINQIKGKISTLLRVRGI 173

SipA_Streptococcus_oralis_Uo5 QLFVLGDNRTTAVDSRAFGTIPIQDTHGKVVTVLRRRGF 183

LepB-Streptococcus-mitis-ATCC6 QLFVLGDNRTTAVDSRAFGTIPIQDTHGKVVTVLRRRGF 183

LepB_Streptococcus_sanguinis_A QLFVLGDNRTTAVDSRAFGTIPIQDTHGKVVTVLRRRGF 183

Streptococcus_pneumoniae QLFVLGDNRTTAVDSRAFGTIPIQDTQGKVVTVIRRRGF 183

S. pyogenes M1 SYLILNDYYSQESENYLQDAIAKDAIKGTINTLIRLRNH 185

::*.* :. .::. : :*.: *::* *.
