# Supplementary material for: Structure and Activity of Streptococcus pyogenes SipA: A Signal Peptidase-Like Protein Essential for Pilus Polymerisation
Source: PLoS One. 2014 Jun 9;9(6):e99135. doi: 10.1371/journal.pone.0099135 (PMC4049620; doi:10.1371/journal.pone.0099135)
Supplement: Table S1 — PCR Primer details. (DOC) [file pone.0099135.s005.doc]

1. Primer Table

| **Primer Name** | **Primer Sequence** |
| --- | --- |
| **T9 SipA cloning** | |
| **SPY0127-F1** | 5'- AAA *GGCGCC* CAG TAT GTT TTT GGT GTT ATG ATT A -3’ |
| **SPY0127-R1** | 5’- AAA *GAATTC* TTA AAT TCC TCT CAC TCT TAA TAG AGT TGA G -3’ |
| **Construction of PilM1 operon** | |
| **PilM1 Bam-F** | 5'-GC *GGATCC* GAT ATG ATG TCA CAT TGA GAG -3’ |
| **M1SipA del-R** | 5'-AAA *CTCGAG* TTA GAA GTT CTG AAA TCA GCT C -3’ |
| **M1SipA del-F** | 5'-AAA *CTCGAG*TTAA*GCGGCCGC* TAA AGG CTT ATT TAA AAA AGG -3’ |
| **PilM1 SalI-R2** | 5'-AAA *GGCGCC GTCGAC* GTC GTG GGG CAA TAA AAA ATT C -3’ |
| **PilM1 SipA-F** | 5’-TAA *CTCGAG* TGG AGA ACT TAA CAA ACT ATC AT -3’ |
| **PilM1 SipA-R** | 5'-AAA *GCGGCCGC* TTA ATG GTT TCT TAA TCG AAT AAG TG -3’ |
| **M1/T1 SipA mutagenesis** | |
| **M1SipA D61A-F2** | 5'- **℗-GCT** ATG GCT CCT AGT GTA AAA GCA G -3’ |
| **M1SipA D61A-R2** | 5'- **℗-**GTT TCT TTT GAT AAT CAT AAA TCC -3’ |
| **M1SipA D61S-F2** | 5'- **℗-TCT** ATG GCT CCT AGT GTA AAA GCA G -3’ |
| **M1SipA K98A-F2** | 5'- **℗-GCA** GTC GGT CGG ATT ATT GCT CAA G -3’ |
| **M1SipA K98A-R2** | 5'- **℗-**CGT TAT AGA TGT TTT AGA GTC CTC -3’ |
| **M1SipA V99R-F1** | 5'- **℗-**AAA **CGT** GGT CGG ATT ATT GCT CAA GCA GGT GAT G -3’ |
| **M1SipA V99R-R1** | 5'- **℗-**CGT TAT AGA TGT TTT AGA GTC CTC -3’ |
| **M1/T1 operon with T9 SipA** | |
| **T9SIPA-F1** | 5'-AAA*CTCGAG*T ATG ACT AAT TAC CTG AAT CGT TTA -3’ |
| **T9SIPA-R1** | 5'-AAA *GCGGCCGC* TTA AAT TCC TCT CAC TCT TAA TAG AGT TGA G-3’ |

Restriction endonuclease sites shown in italics

**℗- =** 5'-phosphorylation

Bold nucleotides show mutated codons
