# Supplementary material for: Structure and Activity of Streptococcus pyogenes SipA: A Signal Peptidase-Like Protein Essential for Pilus Polymerisation
Source: PLoS One. 2014 Jun 9;9(6):e99135. doi: 10.1371/journal.pone.0099135 (PMC4049620; doi:10.1371/journal.pone.0099135)
Supplement: Table S2 — SAX data-collection and scattering-derived parameters. (DOC) [file pone.0099135.s006.doc]

1. SAX data-collection and scattering-derived parameters

| **Data-collection parameters** |  |
| --- | --- |
| Instrument | SAXS/WAXS beamline, Australian Synchrotron |
| Camera length (mm) | 3400 |
| Wavelength (Å) | 1.0332 |
| Beam geometry | 120 micron point source |
| *s* range (Å^-1^) | 0.0007-0.0341 |
| Exposure time | 10 x 1sec |
| Concentration range (mg ml^-1^) | 1-20 |
| Temperature (K) | 293 |
| **Structural parameters** |  |
| *I*(0) (cm^-1^) [from *P*(*r*)] | 0.138 ± 0.001 |
| *R*_g_ (Å) [from *P*(*r*)] | 36.97 ± 0.05 |
| *I*(0) (cm^-1^) (from Guinier) | 0.138 ± 0.000 |
| *R*_g_ (Å) (from Guinier) | 36.88 ± 0.20 |
| *D*_max_ (Å) | 112 |
| Porod volume estimate (Å^3^) | 325171 |
| Molecular-mass determination |  |
| Partial specific volume (cm^3^ g^-1^) * | 0.724 |
| Contrast (Δρ x 10^10^ cm^-2^) | 2.18 |
| Molecular mass *M*_r_ [from *I*(0)] (kDa) | 153.0 |
| Calculated *M*_r_ from sequence (kDa) | 148.9 |
| **Programs used** |  |
| Primary data reduction | *SAXS15*1D |
| Data processing | *PRIMUS, GNOM* |
| Evalution of scattering intensities | *CRYSOL* |

* = (Mylonas and Svergun, 2007)
